# Skeletal Muscle Mass Reduction Velocity as a Simple Prognostic Indicator for Patients with Metastatic Urothelial Carcinoma Receiving Second-Line Chemotherapy

**DOI:** 10.31557/APJCP.2019.20.10.2995

**Published:** 2019

**Authors:** Takashi Nagai, Taku Naiki, Keitaro Iida, Satoshi Nozaki, Toshiki Etani, Yosuke Sugiyama, Ryosuke Ando, Takahiro Yanase, Ryosuke Chaya, Yoshinobu Moritoki, Daichi Kobayashi, Hidetoshi Akita, Takehiko Okamura, Takahiro Yasui

**Affiliations:** 1 *Department of Nephro‐Urology, Nagoya City University, Graduate School of Medical Sciences, *; 3 *Department of Pharmacy, Nagoya City University Hospital, Nagoya,*; 2 *Department of Urology, Anjo Kosei Hospital, Anjo City, Japan. *

**Keywords:** Metastatic Urothelial Carcinoma, second-line chemotherapy, prognostic factor, sarcopenia

## Abstract

**Background:**

Patients with metastatic urothelial carcinoma (mUC) have an uncertain prognosis. The aim of the current study was to evaluate the prognostic potential of a skeletal muscle mass reduction index measured by computed tomography (CT) for mUC patients undergoing second-line gemcitabine and docetaxel (GD) chemotherapy.

**Methods:**

We retrospectively reviewed 44 patients with mUC who received second-line GD chemotherapy between 2006 and 2015 in our hospital. Skeletal muscle area (SMA) at the third lumbar vertebra was measured using CT images obtained from medical records, and a skeletal muscle index (SMI) was calculated for each patient as: SMI = SMA / height2. Changes in SMI across timepoints (SMI inclination) were calculated as: SMI inclination = [( SMI/SMI)/duration of the interval between imaging visits]. Patients were then divided into two groups: a “steep” group (SMI inclination < -0.01) and a “gentle” group (SMI inclination ≥ -0.01). Kaplan-Meier curves and multivariate Cox proportional hazards regression models were used to evaluate the relationship between SMI inclination and overall survival (OS).

**Results:**

There were no differences in patient characteristics between the two groups with respect to median age, gender, Eastern Cooperative Oncology Group Performance Status (ECOG-PS), disease control rate or first-line treatment regimen. OS from the start of second-line GD therapy group was significantly shorter in the “steep” group relative to the “gentle” group. The multivariate analysis revealed that “steep” SMI inclination and presence of anemia were strong predictors of poor prognosis.

**Conclusion:**

Higher values of SMI inclination, indicating a faster rate of skeletal muscle mass reduction, may serve as a useful predictive marker for OS in mUC patients undergoing second-line GD chemotherapy.

## Introduction

The standard treatment for metastatic urothelial carcinoma (mUC) is cisplatin-based chemotherapy. The National Comprehensive Cancer Network recommends dose-dense combination chemotherapy regimens of methotrexate, vinblastine, doxorubicin, and cisplatin (MVAC), or a combination of gemcitabine and cisplatin (GC) for patients who are cisplatin eligible (Spiess et al., 2017). The overall 5-year survival rate is reported as 6.8% (von der Maase et al., 2005). As such, advances in the therapeutic approach are necessary in order to improve prognosis after a diagnosis of mUC.

Several second-line therapies have been examined for use in patients with mUC, including immunotherapy after failure of platinum-based chemotherapy. We previously reported on the use of gemcitabine and docetaxel (GD) combination therapy in patients with mUC (Naiki et al., 2014). Subsequent to that report, in 2016, the PD-L1 antibody atezolizumab was approved by the FDA (Rosenberg et al., 2016), making it the sixth different monoclonal antibody FDA-approved for treating patients who failed on first-line platinum-based treatments for advanced/metastatic disease. Immune checkpoint inhibition is well tolerated, even among elderly and comorbid patients, and shifts the treatment paradigm from initial response to long-lasting disease stabilization, which is achieved in approximately 20% of patients. As such, immunotherapy shows promise as a second-line treatment for mUC. In order to show the clinical utility of GD therapy as a second-line treatment for mUC, a plausible argument needs to be made for the use of toxic chemotherapy. One potential line of reasoning is that the field lacks predictive biomarkers that can be used for determining which therapies are most likely to be effective in individual patients. Although several studies have supported the efficacy of immunotherapy, not all studies could demonstrate a definitive improvement in prognosis, as only 30% of patients actually benefit from immunotherapy. This leads to the obvious clinical question, “which patients will benefit from second-line chemotherapy, and which patients will respond to second-line immunotherapy?”. However, at present there are no data available to investigate this query. Similarly, there is a lack of data that could be useful for therapeutic decision-making, such as whether to continue a second-line therapy, switch to a new therapy, or begin best supportive care. Although new biomarkers are constantly being developed, it is difficult to identify markers that are both easy to use and strongly correlated with a useful clinical endpoint. No such definitive biomarker is available to predict prognosis in patients with mUC. 

Sarcopenia, or reduction of skeletal muscle, is categorized into two groups by the European Working Group on Sarcopenia in Older People: primary sarcopenia, which is caused by aging; and secondary sarcopenia, which is caused by malnutrition, disease, and a decline in physical activity (Cruz-Jentoft et al., 2010). Although evaluations of sarcopenia are not yet standardized, estimating muscle mass according to computed tomography (CT) has been advocated for recently. Sarcopenia diagnosed by CT has been previously reported to be a useful biomarker for predicting mortality (Fukushima et al., 2015) and postoperative complications (Miyake M et al., 2017) in UC patients. Sarcopenia diagnosed by CT has also been reported to be an independent predictor of poor prognosis among patients with mUC who underwent first-line systemic chemotherapy (Taguchi et al., 2016). 

Although sarcopenia has been documented as a significant prognostic factor, to our knowledge, there are no previous studies examining the association between GD chemotherapy as a second-line treatment for mUC, and the presence or extent of sarcopenia. In this study, we analyzed sarcopenia progression by measuring the rate of skeletal muscle mass reduction using CT imaging, and found that skeletal muscle reduction speed during GD chemotherapy was a valuable indicator of prognosis.

## Materials and Methods

*Patient enrollment*

We retrospectively reviewed patient records to identify subjects with mUC who had received cisplatin-based chemotherapy as a first-line treatment and GD chemotherapy as a second-line treatment between 2006 and 2015 in our hospital. A total of 49 patients were identified, although 5 patients were excluded due to a lack of CT data, leaving a final study population of 44 patients. An axial CT was obtained at the onset of GD chemotherapy, and during two subsequent courses of GD chemotherapy. The median time between the start date of GD chemotherapy and the date of completing 2 courses of GD chemotherapy was 2.7 months (interquartile range (IQR), 1.3-5.9 months). 

Patients were allocated into two groups according to skeletal muscle index (SMI, see below) inclination: a steep group (SMI inclination <-0.01) and a gentle group (SMI inclination ≥-0.01). We then examined the relationship between SMI inclination and overall survival (OS). 

*Treatment schedule*

The 44 selected patients were given an intravenous infusion of gemcitabine (800 mg/m^2^) for 30 min and docetaxel (40 mg/m^2^) for 60 min on days 1 and 8, as per a previously published protocol (Dreicer et al., 2003). This cycle was repeated every 21 days. Dexamethasone (6.6 mg administered intravenously for 30 min) was used as premedication for docetaxel. The same gemcitabine and docetaxel doses were administered on days 1 and 8 of each cycle if patients maintained white blood cell (WBC) and platelet counts > 3,000 and > 75,000 µL/mL, respectively. Treatment was discontinued in each cycle if the counts were lower than these thresholds. When grade 3 adverse events (AEs) occurred, a 10% dose reduction was applied in the next cycle, and GD treatment was continued until progression. The efficacy of the GD regimen as a second-line chemotherapy was assessed in a follow-up analysis. Anti-emetics and analgesics were given as supportive care to patients experiencing AEs. All patients were evaluated for the presence of any toxicity, and were assessed at every cycle with imaging by enhanced CT. Patients were required to have an Eastern Cooperative Oncology Group performance status (ECOG–PS) of 1 or lower as per World Health Organization (WHO) criteria: an adequate bone marrow reserve (WBC count > 3,500/µL, platelet count > 100,000/µL, and hemoglobin > 10 g/dL). Other requirements included: reasonable hepatic function (serum bilirubin ≤ 1.5 mg/dL), and an estimated life expectancy ≥ 12 weeks. Clinical response was assessed based on revised Response Evaluation Criteria in Solid Tumors (RECIST) guideline (RECIST ver. 1.1). (Eisenhauer EA et al., 2009) Disease control rate is defined as percentage of patients who have achieved complete response, partial response and stable disease. Prognostic comorbidity was estimated using the Charlson comorbidity index (CCI) (Charlson ME et al., 1987). The institutional chemotherapy review boards (ethical committees) of Nagoya City University Hospital and Nagoya City University (#1152) approved this study, which was conducted in accordance with the Declaration of Helsinki (according to the 2004 Tokyo revision).

**Table 1:** 

Parameters	Steep group	Gentle group	P-value
	(n=16)	(n=28)	
Median age (range), years	70 (50-80)	65 (41-82)	n.s.
Gender, n (%)			
Male	12 (75.0)	21 (75.0)	n.s.
Female	4 (25.0)	7 (25.0)	
ECOG-PS, n (%)			
0	6 (37.5)	18 (64.3)	n.s.
1	8 (50.0)	7 (25.0)	
2	2 (12.5)	3 (10.7)	
First line chemotherapy regimen, n (%)			
MVAC	1 (6.3)	1 (3.6)	
GC	8 (50.0)	17 (60.7)	n.s.
GEM+CBDCA	4 (25.0)	0 (0)	
MEC	3 (18.7)	10 (35.7)	
Disease control rate, %	50	71.4	n.s.
Median SMI at initiation of second-line GD therapy (range), cm^2^/m^2^	40.9 (26.1-49.2)	38.0 (26.4-51.5)	n.s.
Median SMI inclination (range)	-0.019	0.0073	<0.01**
	(-0.011 – 0.089)	(-0.0099 – 0.07)	
Median Survival Time, Months	5.4	10.7	<0.01**

ECOG-PS, Eastern Cooperative Oncology Group Performance Status; MVAC, Methotrexate, Vinblastine, Adriamycin, Cisplatin; GC, Gemcitabine, cisplatin; MEC, Methotrexate, Etoposide, Cisplatin; GD, Gemcitabine, Docetaxel; SMI, Skeletal muscle index

**Figure 1:**
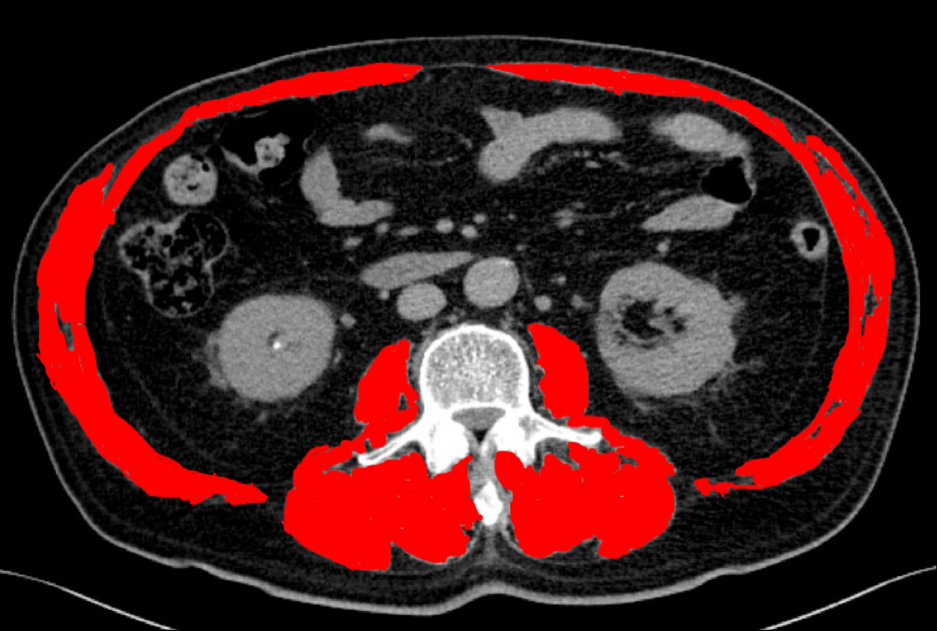
Axial CT Showing SMA at L3. Total area of iliopsoas, erector spinae and abdominal wall muscle are count as SMA (red area).

**Figure 2:**
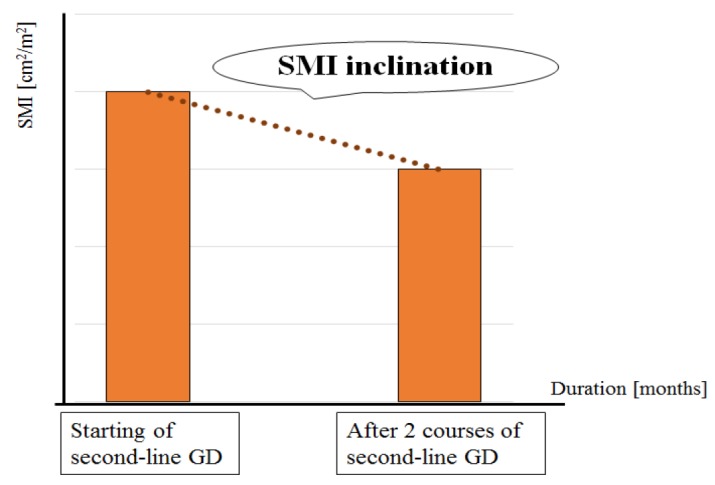
SMI Inclination is Defined as the Rate of Change in SMI Over the Duration between Imaging Visits. In this study, the second visit took place after two courses of second-line GD chemotherapy

**Figure 3:**
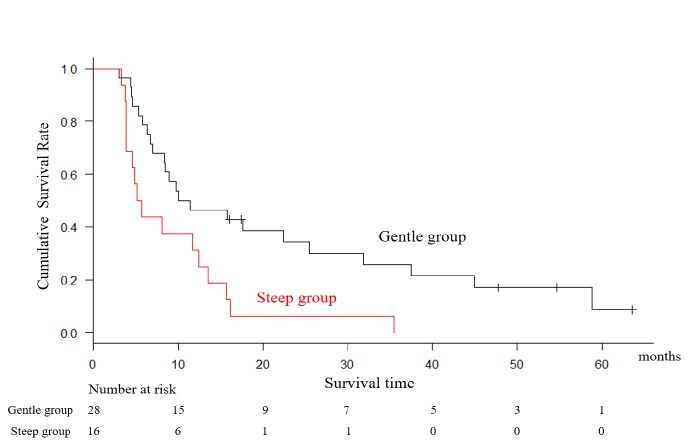
Kaplan-Meier Log Rank Analysis Showed that Patients in the “Steep” Group (SMI Inclination < -0.01) had Poorer OS, Measured from the Start of Second-Line GD Chemotherapy, Compared with Those in the “Gentle” Group (5.4 versus 10.7 months, log-rank p-value < 0.01)

**Table 2:** Multivariable Analysis Potential Prognostic Fctors, with OS as the Outcome

Variables	HR	95% CI	P value
Age below/above the median (≤ 67 vs. ≥ 68 years)	0.9	0.42-1.96	n.s.
Gender (male vs. female)	1.74	0.69-4.40	n.s.
Changes in ECOG-PS (stable vs. deterioration)	1.58	0.68-3.66	n.s.
CCI (≤ 7 vs. ≥ 8)	1.04	0.44-2.44	n.s.
Disease control (CR, PR and SD vs. PD)	1.12	0.39-3.20	n.s.
SMI inclination (steep vs. gentle)	2.38	1.05-5.38	0.037
Hb at starting GD below/above (≥ 10 vs. <10 g/dl)	2.23	1.00-5.00	0.049
Changes in body weight below/above the median (≥ -1.35 vs. < -1.35 kg)	1.16	0.51-2.63	n.s.
Liver metastasis (absence vs. presence)	0.96	0.15-6.29	n.s.
Time from starting 1st line chemotherapy below/above the median (< 202 vs. ≥ 202 days)	1	1.00-1.00	n.s.

OS, Overall survival; HR, Hazard ratio; CI, Confidence interval; ECOG-PS, Eastern Cooperative Oncology Group Performance Status; CR, Complete response; PR, Partial response; SD, Stable disease; PD, Progressive disease; CCI, Charlson comorbidity index; SMI, Skeletal mass index; GD, Gemcitabine and docetaxel

*Imaging analysis and interpretation*

Skeletal muscle area (SMA) at the third lumbar vertebrae (L3) was measured using CT imaging. Skeletal muscle at L3 includes areas of iliopsoas muscle, erector spinae muscle, and muscle of the abdominal wall. Figure 1 shows an axial CT indicating SMA at L3. SMA was measured by a trained urologist using specialized imaging software. Based on the value of SMA, a skeletal muscle index (SMI) was calculated for each patient according to the following formula: 

$\mathrm{SMI}=\frac{\mathrm{SMA}}{\left(\mathrm{height}\right)2}$                      (expressed as cm^2^/m^2^). 

Additionally, we developed a novel index to represent the speed of reduction in skeletal muscle: the SMI inclination. SMI inclination is the rate of change in SMI over the duration (in months) between patient visits and is defined as: 

$\mathrm{SMI inclination}=\frac{\mathrm{⊿SMI}/\mathrm{SMI}}{\mathrm{duratuion}}$                        (Figure 2). 

*Statistical analysis*

Differences in categorical parameters were assessed using Fisher’s exact test, Student’s t-test, or the chi-square test, as appropriate. Cumulative rates were estimated using the Kaplan-Meier method, and differences between curves were evaluated using the log-rank test. Univariate and multivariate analyses were conducted using the Cox proportional hazard regression model. A value of p < 0.05 was considered statistically significant. All data were analyzed using EZR software (Saitama Medical Center, Jichi Medical University, Yakushiji, Japan).

## Results

This study included data from 44 mUC patients. A total of 39 patients died of UC during the follow-up period, and the median survival time was 9.38 months (Table 1). The median age at onset of GD therapy was 67.5 years, 33 patients (75%) were men and 11 (25%) were women. The median SMI at GD onset was 38.9 cm2/m2 (IQR, 26.1-51.5 cm^2^/m^2^). The median SMI inclination during 2 courses of GD was -0.0012 (IQR, 26.1-51.5 cm^2^/m^2^). There were no significant differences between the steep group and the gentle group with regard to patient parameters collected at baseline, including gender, ECOG-PS, SMI, and type of first-line chemotherapy. 

The Kaplan-Meier log-rank analysis showed that patients in the steep group (SMI inclination < -0.01) had poorer OS (measured from the date of initial GD therapy) than those in the gentle group (5.4 versus 10.7 months, log-rank p-value < 0.01; Figure 3). Multivariable Cox proportional hazards regression was used to evaluate the association between OS and SMI inclination, as well as other patient parameters, including age, gender, changes in ECOG-PS, CCI, disease control, Hb at starting GD, changes in body weight, liver metastasis, time from starting 1^st^ chemotherapy. These analyses demonstrated that “steep” SMI inclination (HR, 2.38; 95% CI, 1.05-5.38, p=0.037) and presence of anemia (HR, 2.23; 95% CI, 1.00-5.00, p=0.049) were significant predictor of worse prognosis (Table 2).

## Discussion

The results of this study indicate that SMI inclination offers promise as a potential prognostic marker among mUC patients undergoing GD as second-line chemotherapy. To our knowledge, this is the first evaluation of the association between speed of sarcopenia progression and prognosis. Although platinum-based agents as first-line chemotherapy bring benefits for mUC patients, significant numbers of UC patients are cisplatin-unfit due to age, comorbidities, or a history of nephrectomy. Moreover, platinum-based chemotherapy causes degeneration in renal function. However, as we have previously reported, GD therapy is well-tolerated and can be safely used in patients previously treated for impaired renal function (Naiki et al., 2014). For mUC patients who are cisplatin-unfit, GD therapy brings some level of benefit. However, there are no definitive biomarkers available that can be used as guidance for determining which mUC patients will be most likely to benefit from GD therapy. 

Sarcopenia reflects multiple facets of cancer cachexia and can be used as an indicator of cancer aggressiveness, as sarcopenia has been shown to be an independent predictor of OS among advanced UC patients (Fukushima H et al., 2015). Measurement of SMI based on CT has also been reported as an independent predictor of poor prognosis among mUC patients who underwent first-line systemic chemotherapy (Taguchi et al., 2016). Although many of these patients had already developed sarcopenia prior to chemotherapy, progression of sarcopenia, namely, the speed of reduction in skeletal muscle, was not examined. In the current study, there was a significant difference in OS according to SMI inclination, but there was no difference in OS according to the single measure of SMI obtained at the onset of GD therapy. Additionally, in previous reports involving mUC patients, the method of treatment was not uniform across all patients. In contrast, patients enrolled in the current study received various platinum-based chemotherapeutic agents in the first-line setting, but the second-line chemotherapy was uniform. 

Since we developed SMI inclination as a novel prognostic marker, there is no established cutoff value available from past literature. The typical reduction of skeletal muscle is 5% per decade, beginning at 30 years of age, and the ratio accelerates among people older than 65 years (Lexell et al., 1988). The diagnostic criteria for cachexia, as defined by the European Palliative Care research collaborative, are: a) weight loss greater than 5% over 6 months; or b) weight loss greater than 2% in individuals with a body-mass index <20 kg/m^2^; or c) weight loss greater than 2% and sarcopenia (Fearon, 2011). We set the cutoff for speed of reduction in skeletal muscle index at 1% per month. As SMI inclination is a novel parameter, additional data will be required in order to determine the optimal cutoff value. 

The use of CT imaging is convenient and straightforward for patients because CT images are already used at regular intervals for evaluating response to treatment. As such, measuring SMI inclination requires no extra procedures or additional costs for the patients. As SMI inclination is calculated after 2 courses of GD therapy, it is impossible to predict prognosis before treatment. But measuring the rate of reduction in the skeletal muscle index based on CT during the first 2 courses of chemotherapy may provide an early indication of treatment efficacy. It is a feasible way to determine whether continuing chemotherapy is likely to be beneficial for the patient. 

We conducted multivariate analysis including these factors based on previous reports. There are some reports regarding the prognosis of predictive factor. Favorable PS, female gender, hemoglobin level > 10 g/dl, and single organ metastasis were previously reported to be favorable prognostic factors among Japanese mUC patients treated with systemic chemotherapy (Abe et al., 2016). Moreover, the ECOG PS at presentation, CRP level and response to prior chemotherapy were reported as prognostic factors for mUC patients undergoing second-line chemotherapy (Matsumoto et al., 2018). In the setting of second-line chemotherapy, time from prior chemotherapy, alkaline phosphatase, visceral involvement and liver metastasis are also reported as worse prognosis of predictive factor (Bellmunt et al., 2009, Sonpavde et al., 2013). Regarding CCI, this method is intended for estimating risk of death from comorbid disease (Charlson et al. 1987). Although previous reports have suggested that it is a useful prognostic marker, it was not found to be predictive of OS in this study. This may be due to the scoring system, which assigns 6 points if the patient has a “metastatic solid tumor”. All the patients in this study met that criteria, such that CCI was always above 6 points, leaving little space for heterogeneity and no obvious method for dividing patients according to CCI. In this study, PS deterioration and clinical response were not shown to be a significant predictive factor in the multivariable analysis. They are considered as prognostic factors but they have some problems to evaluate conditions of patients. Assessment of PS depends on each physician and considered as subjective factor. Moreover, changes in PS is scored in the range of 1 - 4, it is difficult to make delicate difference. The reason of clinical response was not shown to be a significant predictive factor was considered that evaluation of clinical response during 2 courses are too early to predictive prognosis. 

The main limitations of our study were the retrospective design, the small sample size, and the short duration of follow-up. Additionally, there is no established cutoff value for SMI inclination, as this a novel prognostic indication. Further studies with larger population are needed to confirm our findings.

In conclusion, higher values of SMI inclination, indicating a faster rate of skeletal muscle mass reduction, may serve as a useful predictive marker for OS in mUC patients undergoing second-line GD chemotherapy.
